# Inhibitory Effect of Sirtuin6 on EndMT by Regulating Oxidative Stress and Autophagy in Coxsackievirus B3‐Induced Cardiac Endothelial Cells

**DOI:** 10.1002/iid3.70316

**Published:** 2025-12-29

**Authors:** Zimei Yang, Yimin Wang, Jun Chen, Xinyang Shou, Di Zhang, Zhidong Zhou, Qiang Liu

**Affiliations:** ^1^ The Third Affiliated Hospital of Zhejiang Chinese Medical University Hangzhou Zhejiang China; ^2^ Department of Cardiology Zhejiang Provincial People's Hospital (Affiliated People's Hospital, Hangzhou Medical College) Zhejiang China

**Keywords:** autophagy, coxsackievirus B3, endothelial‐to‐mesenchymal transition, oxidative stress, Sirtuin6

## Abstract

**Objective:**

Sirtuin 6 (Sirt6) plays a critical role in cardiovascular pathophysiology, yet its involvement in viral myocarditis (VMC) remains poorly understood. This study aimed to investigate the role of Sirt6 in coxsackievirus B3 (CVB3)‐induced endothelial‐to‐mesenchymal transition (EndMT) and its underlying molecular mechanisms.

**Methods:**

A model of CVB3‐infected mouse cardiac endothelial cells (MCECs) was established. EndMT markers and Sirt6 expression were detected by WB/IF and qRT‐PCR. Lentivirus‐mediated Sirt6 knockdown or overexpression was performed to examine its impact on EndMT. Apoptosis and apoptosis‐related proteins were analyzed by flow cytometry and WB. Proteomic analysis was further conducted on Sirt6‐knockdown MCECs and their controls. Based on the results, oxidative stress and autophagy were assessed in CVB3‐induced EndMT, and the influence of altered Sirt6 expression on these indicators was evaluated.

**Results:**

Sirt6 expression was significantly downregulated in CVB3‐induced EndMT. Sirt6 knockdown promoted EndMT, as manifested by decreased vascular endothelial cadherin (VE‐cad) and increased α‐smooth muscle actin (α‐SMA) expression. It also exacerbated apoptosis, accompanied by upregulation of pro‐apoptotic Bax, downregulation of anti‐apoptotic Bcl‐2, and an increase in Caspase‐3 expression. Sirt6 overexpression partially reversed these changes. Proteomic analysis indicated that Sirt6 was involved in inflammatory signaling, apoptotic cascades, redox homeostasis, and metabolic pathways. CVB3 infection markedly elevated intracellular oxidative stress (increased ROS and MDA levels, decreased SOD activity) and suppressed autophagy (reduced LC3B‐II and Beclin‐1, elevated p62). These CVB3‑induced effects were aggravated by Sirt6 knockdown but attenuated by Sirt6 overexpression.

**Conclusion:**

This study reveals that Sirt6 inhibits CVB3‐induced EndMT by regulating oxidative stress and autophagy. These findings provide experimental evidence for elucidating the pathological mechanisms of VMC and suggest Sirt6 as a potential therapeutic target.

## Graphical abstract

This study found that Sirtuin 6 can inhibit CVB3‐induced endothelial‐to‐mesenchymal transition. This protective effect involves the regulation of oxidative stress and autophagy, providing new experimental evidence for elucidating the pathological mechanisms of viral myocarditis.

## 1 Introduction

Viral myocarditis (VMC) is an inflammatory cardiac disease primarily initiated by viral infections, with enteroviruses‐especially Coxsackievirus B (CVB)‐being the most common causative agents, along with adenovirus and influenza virus [1]. A critical early step in VMC pathogenesis involves the breaching of the cardiac microvascular endothelial barrier by circulating CVB to reach and infect cardiomyocytes [2, 3]. Importantly, the pathological role of CVB extends beyond VMC, as evidenced by its significant association with acute myocardial infarction (MI). Serological studies reveal higher detection rates of CVB‐specific antibodies and viral RNA in MI patients vs. controls, suggesting that CVB contributes to both inflammatory and ischemic cardiac injury [4, 5].

CVB induces cardiac damage through dual mechanisms. Directly, CVB‐encoded 2A protease cleaves dystrophin's hinge 3 region, disrupting sarcolemmal integrity and increasing myocyte permeability. Viral persistence is enhanced by genomic variations like 5′‐UTR deletions. Indirectly, CVB triggers a “cytokine storm” with elevated TNF‐α, IL‐6, and IL‐1β, reducing contractility and promoting apoptosis, while recruiting immune cells that mediate autoimmune damage to uninfected myocardium [6, 7]. Notably, endothelial‐to‐mesenchymal transition (EndMT) has recently emerged as a critical process in VMC‐related endothelial dysfunction. EndMT represents a phenotypic shift in endothelial cells under pathological conditions—such as viral infection or inflammatory cytokine exposure‐marked by downregulation of endothelial markers (e.g., VE‐cad and Claudin‐5) and upregulation of mesenchymal markers (e.g., α‐SMA, Vimentin, and Fibronectin). This transition enhances cell migration and invasiveness, contributing to microvascular fibrosis, dysfunction, and pro‐inflammatory microenvironment formation processes that collectively aggravate cardiovascular disease progression [8, 9]. Nevertheless, the regulatory mechanisms governing EndMT during CVB infection remain largely unknown.

The sirtuin family comprises evolutionarily conserved NAD⁺‐dependent deacetylases that regulate essential biological processes such as genomic stability, inflammation, and metabolic homeostasis [10, 11]. Among these, Sirt6‐a nuclear sirtuin enriched in the heart—has attracted increasing attention in cardiovascular research over the past decade [12]. Sirt6‐deficient mice develop severe concentric cardiac hypertrophy and exhibit rapid functional decline under certain genetic backgrounds [13]. Mechanistically, Sirt6 inhibits several pro‐hypertrophic pathways, including IGF/AKT, mTORC, and STAT3 signaling, thereby counteracting pathological cardiac remodeling [14]. It also helps maintain endothelial function by modulating the transcription of genes such as TNFSF4, FOXM1, and NF‐κB, thereby attenuating atherosclerotic progression [15]. Although recent studies suggest that Sirt6 alleviates cardiomyocyte pyroptosis and inflammatory responses in VMC by suppressing Wnt/β‐catenin signaling [16], its role in EndMT during CVB infection has not been explored.

In this study, we established an in vitro model of CVB3‐infected mouse cardiac endothelial cells (MCECs) to investigate whether and how Sirt6 regulates EndMT, aiming to provide new insights into Sirt6‐mediated cardioprotection in VMC.

## 2 Materials and Methods

### 2.1 Cell Culture and Treatment

Mouse cardiac endothelial cells (MCECs; Yuanchuang Bio‐technology, Shanghai) were cultured in DMEM supplemented with 10% FBS and penicillin‐streptomycin (100 U/mL and 100 µg/mL, respectively; Gibco, China). The CVB3 Nancy strain was propagated in HeLa cells, and viral titers were determined via a micro‐method, expressed as TCID₅₀/mL of 10⁷. Before infection, cells were serum‐starved overnight in serum‐free medium, infected with CVB3 for 2 h, and then maintained at 37°C for 72 h prior to analysis.

### 2.2 Lentivirus Construction and Administration

Lentiviral vectors were constructed by GeneChem (Shanghai, China). The GV493 vector was used to carry shRNA targeting mouse Sirt6 (sequence: 5′‐GCATGTTTCGTATAAGTCCAA‐3′), while the GV492 vector was used to carry the full‐length mouse Sirt6 cDNA amplified from cardiac tissue. Lentiviral particles were produced using psPAX2 and pMD2.G packaging plasmids. For infection, cells were seeded in six‐well plates at 1 × 10⁵ cells/well. After adherence, the virus solution and infection enhancer were added to the complete medium at an MOI of 100. Following 12 h of infection, the medium was replaced with fresh complete medium. Infection efficiency was assessed approximately 72 h post‐infection using an inverted fluorescence microscope (ZEISS, Germany).

### 2.3 Immunofluorescent Staining

Cells were sequentially fixed with 4% paraformaldehyde (10 min), permeabilized with 0.3% Triton X‐100 (30 min), and blocked with 10% goat serum (30 min). Subsequently, they were incubated with a primary antibody overnight at 4°C, followed by a 30‐min incubation with a green fluorophore (FITC)‐conjugated secondary antibody in the dark. After blue‐fluorescent DAPI staining (5 min), fluorescent images were acquired using a fluorescence microscope (ZEISS, Germany) and quantified with ZEN 3.6 software.

### 2.4 CCK8

Following CVB3 infection, the cultures were maintained for 48 h. Cellular viability was then assessed using a CCK‐8 assay kit (Beyotime, China). Specifically, 10 µL of CCK‐8 solution was added to each well containing 100 µL of culture medium. After incubating for 30–60 min, the absorbance at 450 nm was measured with a microplate reader (PerkinElmer, USA).

### 2.5 Apoptosis

Following trypsinization and PBS washing, the cell pellet was resuspended in binding buffer. Cells were stained with Annexin V‐647 and PI working solutions, followed by a 10–15 min incubation on ice in the dark. The experiment included negative control (untreated cells) and single‐stain controls. After adding binding buffer, samples were immediately analyzed on a flow cytometer (Beckman Coulter, USA), with detection wavelengths set at 647 nm for Annexin V‐647 and 617 nm for PI.

### 2.6 Oxidative Stress

Intracellular reactive oxygen species (ROS) were detected with the dihydroethidium (DHE) probe (Beyotime, China), which produces red fluorescence (Ex/Em = 535/610 nm). The DHE stock was diluted 1:1000 in serum‐free medium to 10 µM, and after 30 min of incubation, cells were imaged (ZEISS, Germany). Superoxide dismutase (SOD) activity and malondialdehyde (MDA) content were assessed using respective assay kits (Beyotime, China). Following cell lysis, total protein was quantified by BCA for normalization. SOD and MDA were then measured per the manufacturer's instructions at 450 and 532 nm, respectively, using a microplate reader (PerkinElmer, USA).

### 2.7 Proteomics

Astral‐DIA‐based quantitative proteomic analysis of Sirt6‐knockdown MCECs was performed by Majorbio (Shanghai, China). Upon collection, cells were snap‐frozen in liquid nitrogen and stored at −80°C. The procedure involved total protein extraction, peptide desalting, and mass spectrometric quantification on the Majorbio platform. Raw data were processed and interpreted bioinformatically using the Majorbio Cloud suite (https://cloud.majorbio.com).

### 2.8 RNA Isolation and qRT‐PCR

Total RNA was extracted using TRIzol reagent (Thermo Fisher, Germany) and reverse‐transcribed into cDNA with the PrimeScript RT Reagent Kit (Takara, Japan). qRT‐PCR was performed on an ABI7500 system (Applied Biosystems, USA) using TB Green chemistry (Takara, Japan), and gene expression was quantified via the $2^{‐∆∆C_{t}}$ method. The Sirt6‐specific primers (Sangon Biotech, China) were: forward 5′‐ATGTCGGTGAATTATGCAGCA‐3′ and reverse 5′‐GCTGGAGGACTGCCACATTA‐3′.

### 2.9 Western Blot

Cellular proteins were lysed in ice‐cold RIPA buffer supplemented with 1 mM PMSF (Beyotime, China). After separation by SDS‐PAGE and transfer to PVDF membranes, the membranes were blocked with 5% non‐fat milk for 1 h at room temperature. Subsequently, they were incubated overnight with primary antibodies at 4°C, followed by HRP‐conjugated secondary antibodies for 1 h at room temperature. Signals were detected using a FluorChem Q imaging system (USA) and quantified by densitometry with ImageJ software (Table 1).

**Table 1:** Primary and secondary antibodies used in the study.

Antibody	Species	Supplier	Catalogue number
VE‐cad	Rabbit	Abcam	ab205336
α‐SMA	Rabbit	Abcam	ab124964
Sirt6	Rabbit	Proteintech	13572‐1‐AP
Gapdh	—	Kangchen Bio‐tech	KC‐5G5
Bax	Rabbit	ABclonal	A20227
Bcl‐2	Rabbit	ABclonal	A19693
Caspase‐3	Rabbit	ABclonal	A19654
LC3B	Rabbit	Proteintech	18725‐1‐AP
Beclin‐1	Rabbit	Proteintech	11306‐1‐AP
p62	Rabbit	Proteintech	18420‐1‐AP
Goat Anti‐Rabbit IgG (H + L) HRP	—	Affinity	S0001
Goat Anti‐Rabbit IgG, FITC Conjugated, H + L	—	Biosharp	BL003A

### 2.10 Statistical Analysis

Data are expressed as mean ± standard deviation (SD). Between‐group comparisons utilized Student's *t*‐test or one‐way ANOVA, with statistical significance defined as *p* < 0.05. All analyses were executed in GraphPad Prism 9.0.

## 3 Results

### 3.1 The Expression of Sirt6 During CVB3‐Induced EndMT in MCECs

**Figure 1:**
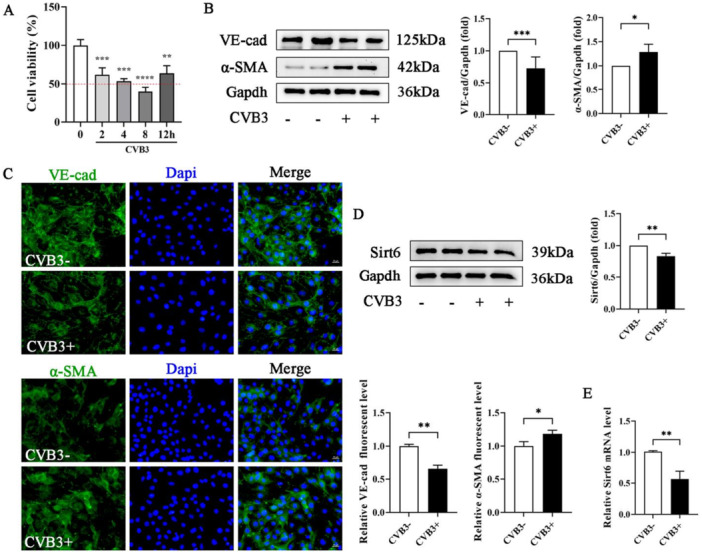
Sirt6 was downregulated during CVB3‐induced EndMT in MCECs. (A) Assay and quantitative analysis of cell viability of MCECs treated with or without CVB3 (represented by CVB3‐ or CVB3 + ) for indicated times. (B and D) Representative blot images and quantitative analysis of EndMT markers and Sirt6 in MCECs. (C) Representative immunofluorescence images and quantitative analysis of VE‐cadherin and α‐SMA in MCECs. The cell nuclei were counterstained with DAPI (scale bar: 20 μm). (E) Detection and quantitative analysis of Sirt6 mRNA levels in MCECs. Data are presented as the mean ± SD of three independent experiments (*n* ≥ 3). *p < 0.05, **p < 0.01, ***p < 0.001.

To establish the optimal infection duration of CVB3 in MCECs, cell viability was quantitatively evaluated across graduated infection time points using CCK‐8 assays. A significant decline in viability was observed between 2 and 12 h post‐infection (Figure 1A). The 2‐h time point was selected as the optimal duration, balancing effective viral infection with maximal cell viability. EndMT was subsequently characterized by molecular marker analysis. Western blot demonstrated significant downregulation of endothelial marker VE‐cad coupled with upregulation of mesenchymal marker α‐SMA in CVB3‐infected MCECs (Figure 1B). Immunofluorescence analysis revealed pronounced attenuation of VE‐cad signals and concomitant enhancement of α‐SMA fluorescence intensity compared with uninfected controls, confirming EndMT progression (Figure 1C). Given the established role of Sirt6 in modulating EndMT through multiple mechanisms, including transcriptional suppression of Malat1, inhibition of Notch1 signaling, and attenuation of vascular inflammation [17, 18, 19], its dynamic expression changes under CVB3 induction were investigated. Both Sirt6 protein and mRNA levels were significantly downregulated in CVB3‐treated MCECs (Figure 1D–E).

### 3.2 The Inhibitory Effect of Sirt6 on CVB3‐Induced EndMT

**Figure 2:**
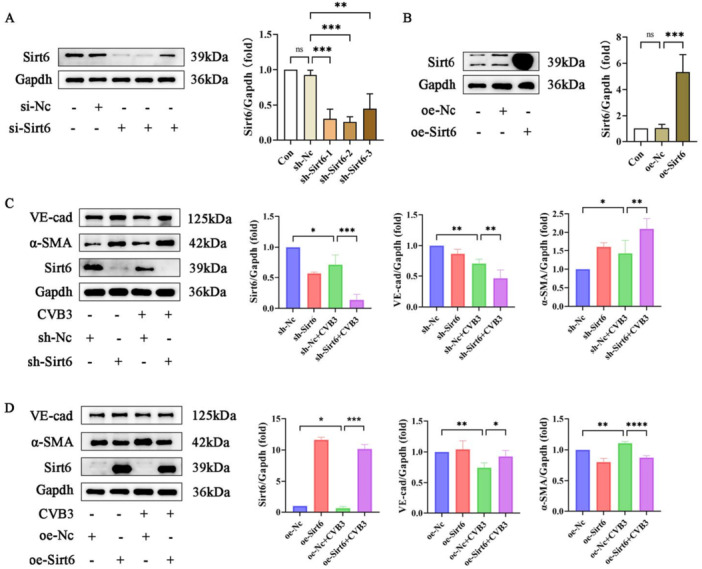
Sirt6 effectively inhibited EndMT to maintain the cell characteristics of MCECs. (A and B) Representative blot images and quantitative analysis of Sirt6 in MCECs with lentiviral transfection. (C and D) Representative blot images and quantitative analysis of Sirt6, α‐SMA and VE‐cadherin in MCECs. Data are presented as the mean ± SD of three independent experiments (*n* ≥ 3). *p < 0.05, **p < 0.01, ***p < 0.001.

To further investigate the regulatory effects of Sirt6 on CVB3‐induced EndMT, lentivirus‐mediated genetic manipulation was employed to establish Sirt6 knockdown and overexpression models. For Sirt6 overexpression, an MOI of 50 was selected due to its superior efficiency compared to MOI 20. For Sirt6 knockdown, the MOI was increased to 100 to achieve satisfactory transfection, as initial attempts at MOI 20 and 50 were suboptimal. Preliminary assessment of transfection efficiency was performed via fluorescence intensity quantification (Supporting Information S1: Figure S1A,B). Western blot analysis confirmed successful modulation of Sirt6 protein expression. Subsequent analysis revealed that the shSirt6‐2 construct exhibited optimal knockdown efficiency, achieving approximately 75% suppression of Sirt6 expression (Figure 2A,B), and was therefore selected for further experiments. Comparative analysis showed significant upregulation of Sirt6 expression in the oe‐Sirt6 group compared with Con and oe‐NC groups (Figure 2A,B). After successful transfection, MCECs were infected with CVB3 for 2 h for phenotypic evaluation. The sh‐Sirt6 + CVB3 group exhibited enhanced phenotypic transformation compared with sh‐NC + CVB3 controls, as evidenced by a significant increase in α‐SMA expression and a further reduced in VE‐cadherin levels (Figure 2C). Conversely, Sirt6 overexpression partially restored endothelial characteristics, effectively suppressing EndMT through upregulation of VE‐cad and downregulation of α‐SMA (Figure 2D). These findings collectively identify Sirt6 as a critical protective modulator against CVB3‐induced EndMT in MCECs.

### 3.3 The Impact of Sirt6 on Apoptosis in MCECs

**Figure 3:**
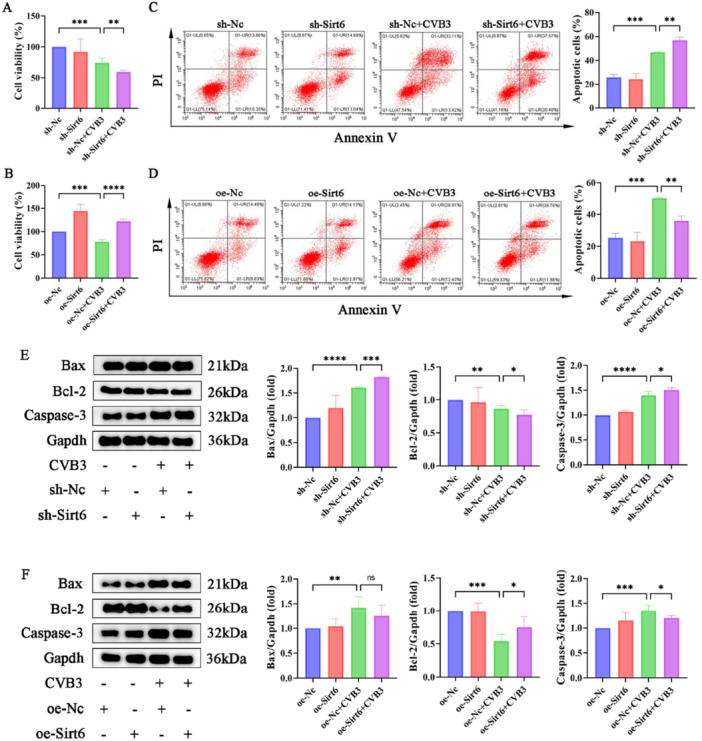
Sirt6 attenuated CVB3‐induced suppression of cell viability and mitigated apoptosis. (A and B) Assay and quantitative analysis of cell viability of lentivirally transfected MCECs with or without CVB3 treatment. (C and D) Representative images and quantitative analysis of cell apoptosis in MCECs. (E and F) Representative blot images and quantitative analysis of apoptosis‐related proteins in MCECs. Data are presented as the mean ± SD of three independent experiments (*n* ≥ 3). *p < 0.05, **p < 0.01, ***p < 0.001.

Previous studies have established that CVB3 replication peaks at 48–72 h post‐infection, after which cells typically undergo pathological alterations, such as proliferation inhibition, apoptosis, and cytolysis [20]. Consistent with these findings, progressive cell death was observed upon prolonged infection in the present study. CCK‐8 assays demonstrated that Sirt6 knockdown exacerbated MCECs viability decline, whereas Sirt6 overexpression attenuated CVB3‐induced cytotoxicity (Figure 3A,B). Subsequent flow cytometry analysis revealed significantly elevated apoptotic rates in sh‐Sirt6 + CVB3 cells compared with sh‐NC + CVB3 controls, a phenomenon that was partially mitigated by Sirt6 overexpression (Figure 3C,D). Pro‐apoptotic Bax and anti‐apoptotic Bcl‐2 act antagonistically, with their expression ratio determining cellular susceptibility to apoptosis [21]. Western blot analysis demonstrated that CVB3‐infected MCECs exhibited upregulated Bax levels and downregulated Bcl‐2 expression, indicating CVB3‐triggered apoptotic activation (Figure 3E,F). Notably, Sirt6 knockdown amplified these expression changes (enhancing Bax upregulation and exacerbating Bcl‐2 suppression), whereas Sirt6 overexpression alleviated CVB3‐mediated apoptotic responses—findings that were consistent with flow cytometry results. To further validate apoptotic progression, Caspase‐3—a key executioner protease in apoptosis—was analyzed [22]. Caspase‐3 levels paralleled Bax expression patterns, suggesting that Sirt6 may exert cytoprotective effects via modulation of Caspase‐3‐dependent apoptotic signaling.

### 3.4 Identification and Functional Enrichment Analysis of Differentially Expressed Proteins

**Figure 4:**
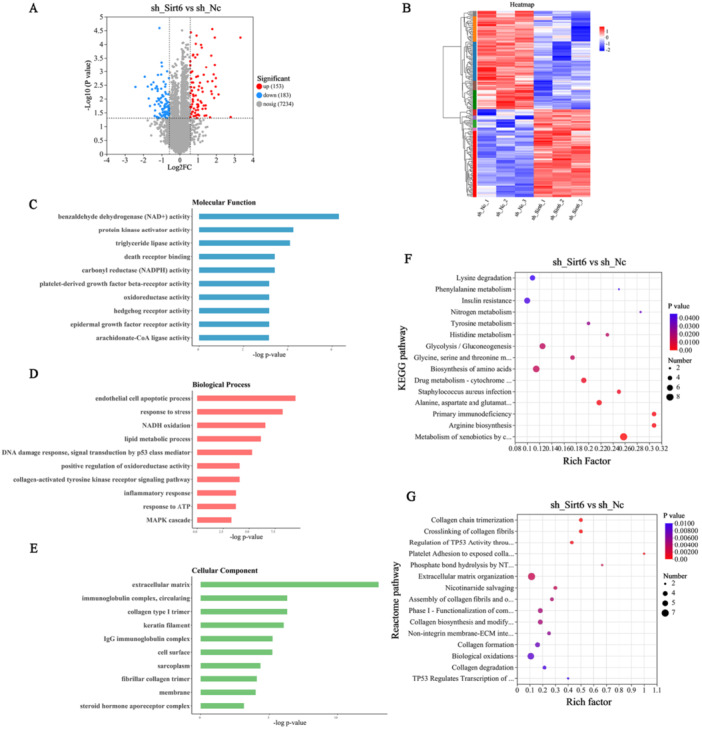
Proteomic profiling revealed DEPs and associated enrichment analyses. (A and B) Volcano and Heatmap plot illustrating DEPs in Sirt6‐knockdown MCECs. (C–E) Bar chart of significantly enriched GO terms. (F and G) Top 15 enriched pathways from KEGG and Reactome analyses.

To elucidate the molecular mechanism underlying Sirt6‐mediated regulation of CVB3‐induced EndMT, proteomic profiling was systematically conducted in Sirt6 knockdown MCECs and control counterparts. Hierarchical clustering analysis revealed distinct expression patterns between experimental groups, where red and blue heatmap gradients represent upregulated and downregulated protein clusters, respectively (Figure 4B). Using selection thresholds (|fold change | > 1.5 and *p* < 0.05), 336 differentially expressed proteins (DEPs) were identified, including 153 upregulated and 183 downregulated proteins (Figure 4A). Multidimensional functional enrichment analyses characterized the biological implications of Sirt6 knockdown. Gene Ontology (GO) analysis revealed significant enrichment in inflammatory signaling pathways, endothelial apoptosis regulation, DNA damage repair processes, and oxidative phosphorylation (Figure 4C–E). Kyoto Encyclopedia of Genes and Genomes (KEGG) pathway analysis demonstrated bidirectional dysregulation of glycolysis/gluconeogenesis, concomitant perturbations in glycine‐serine‐threonine metabolism, histidine degradation pathways, and aromatic amino acid metabolic networks (tyrosine and phenylalanine) (Figure 4F). Reactome pathway analysis identified two core regulatory modules: collagen biosynthesis and biological oxidation cascades (Figure 4G).

### 3.5 The Regulation of Sirt6 on Oxidative Stress and Autophagy in MCECs

**Figure 5:**
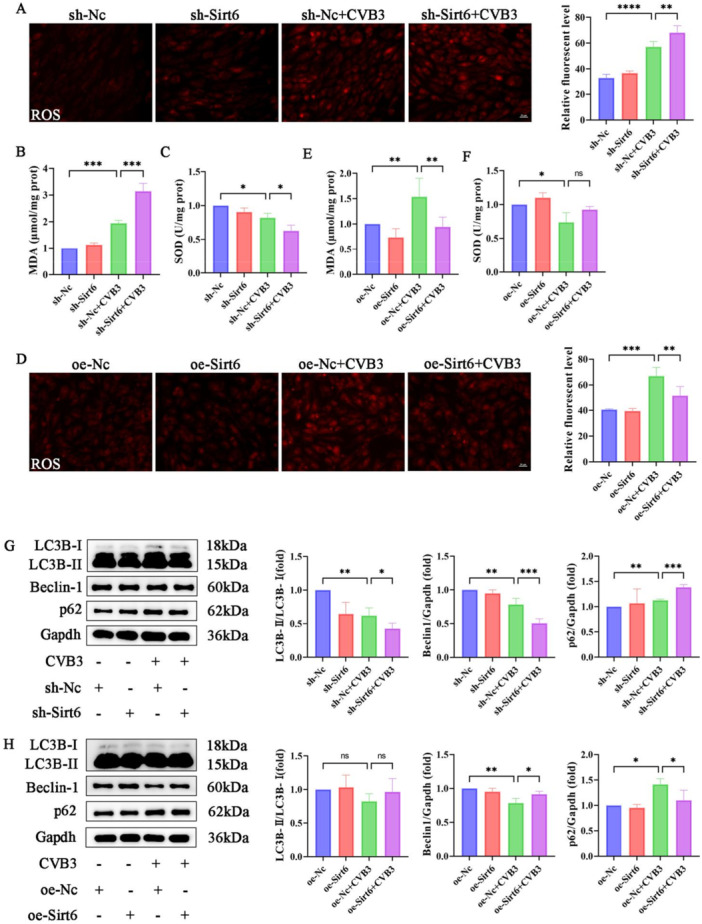
Sirt6 attenuated oxidative stress and restored autophagy. (A and D) Representative fluorescence images and quantitative analysis of ROS in MCECs (scale bar: 20 μm). (B,C and E,F) Assessment and quantitative analysis of MDA and SOD in MCECs. (G and H) Representative blot images and quantitative analysis of autophagy‐related proteins in MCECs. Data are presented as the mean ± SD of three independent experiments (*n* ≥ 3). *p < 0.05, **p < 0.01, ***p < 0.001.

Proteomic analysis revealed a distinct subset of DEPs that were critically involved in maintaining cellular redox homeostasis. Disruption of redox homeostasis triggers excessive production of ROS, whose accumulation induces oxidative stress and ultimately leads to endothelial cell dysfunction [23]. Given this mechanistic link, the regulatory role of Sirt6 in oxidative stress was investigated. In CVB3‐infected MCECs, ROS and MDA levels were significantly elevated, whereas SOD activity was reduced. Sirt6 knockdown exacerbated intracellular oxidative stress, as evidenced by further increases in ROS and MDA levels, accompanied by more pronounced reduction of SOD activity (Figure 5A–C). Conversely, Sirt6 overexpression effectively attenuated these pathological alterations (Figure 5D–F). Existing evidence indicates that excessive ROS serve as signaling molecules to activate autophagy, which in turn reduces ROS generation through clearance of damaged mitochondria and oxidized cellular components, thereby maintaining cellular redox balance [24, 25]. To explore this compensatory mechanism, autophagy‐related markers were analyzed. The data demonstrated decreased LC3B‐II and Beclin‐1 expression, coupled with elevated p62 levels in infected cells, indicating autophagy inhibition. Importantly, the degree of autophagy suppression was negatively correlated with Sirt6 expression levels (Figure 5G,H).

## 4 Discussion

In VMC, endothelial cells form a critical biological barrier that protects cardiomyocytes from pathogen invasion, whereas EndMT represents a central mechanism of endothelial dysfunction [26]. Emerging evidence highlights the pivotal role of Sirt6 in regulating EndMT. Studies have shown that Sirt6 modulates EndMT progression via multiple molecular pathways in response to diverse stimuli. Sirt6 suppresses high glucose‐ or hydrogen peroxide‐induced EndMT by inhibiting the Notch1 and Smad2/3 signaling pathways [17, 27]. In senescence‐associated EndMT, Sirt6 regulates this process by transcriptionally repressing the long non‐coding RNA MALAT1 [18]. In addition, Sirt6 overexpression counteracts cytokine‐induced EndMT [19]. In this study, Sirt6 demonstrated potent inhibition of CVB3‐induced EndMT, as evidenced by significant upregulation of VE‐cad and downregulation of α‐SMA. Furthermore, Sirt6 attenuated CVB3‐triggered apoptotic responses, likely via regulation of Caspase‐3‐dependent apoptotic pathways. This anti‐apoptotic effect is consistent with established mechanisms wherein Sirt6 inhibits Bax mitochondrial translocation through H3K9 deacetylation [28]. Notably, a complex interplay exists between apoptosis and EndMT [29, 30, 31]. On one hand, apoptosis promotes EndMT: apoptotic cells release signaling molecules such as TGF‐β and TNF‐α, which activate EndMT in adjacent endothelial cells. Additionally, apoptosis‐induced microenvironmental changes—such as inflammatory responses and oxidative stress—create a pro‐EndMT milieu. On the other hand, EndMT‐transformed cells acquire anti‐apoptotic properties, facilitating their survival in hostile microenvironments.

Proteomic analysis revealed the crucial regulatory role of Sirt6 in maintaining redox homeostasis, where metabolism‐disordered oxidative stress serves as a key driver of EndMT. Oxidative stress is defined as a pathological imbalance between ROS production and antioxidant defense capacity. Research demonstrates that glycolytic dominance‐induced NADPH deficiency, combined with mitochondrial dysfunction, triggers abnormal ROS accumulation, resulting in sustained oxidative stress and exacerbation of endothelial dysfunction [32, 33]. Previous studies confirm that Sirt6 counteracts oxidative stress via multiple pathways, including ROS reduction [34, 35], antioxidant enzyme regulation [36], and inhibition of pro‐oxidative signaling pathways [37]. For instance, Sirt6 overexpression activates the AMPK pathway, upregulating anti‐apoptotic Bcl‐2 and suppressing NF‐κB activity, which collectively reduce ROS levels and alleviate hypoxia‐induced cardiomyocyte injury [38]. Additionally, Sirt6 protects vascular endothelial cells against angiotensin II‐induced oxidative stress by activating the Nrf2/ARE pathway [39]. In this study, CVB3 infection significantly elevated ROS and MDA levels while suppressing SOD activity, indicating oxidative stress that was effectively mitigated by Sirt6 overexpression. Intriguingly, studies report bidirectional regulatory crosstalk between Sirt6 and Sirt3 [40, 41]. Sirt6 enhances Sirt3 expression by transcriptionally activating nuclear respiratory factor 2, whereas Sirt3 exerts antioxidant effects through mitochondrial deacetylation. However, the present study did not further explore the mechanistic interplay between Sirt6 and Sirt3 during CVB3 infection.

Autophagy, an evolutionarily conserved catabolic pathway, plays essential roles in maintaining cellular homeostasis [42, 43]. ROS, as key mediators of oxidative stress, activate autophagy‐related signaling pathways. Moderate autophagy exerts protective effects by scavenging ROS sources and activating antioxidant pathways, whereas sustained oxidative stress disrupts the autophagy‐redox balance, exacerbating cellular damage [44]. Studies demonstrate that autophagy activation promotes Snail protein degradation in human cardiac microvascular endothelial cells, thereby suppressing epithelial‐mesenchymal transition (EMT) [45]. Previous studies using cardiomyocyte or cardiac fibroblast models reveal that CVB3 infection transiently activates autophagy to facilitate viral replication, followed by lysosomal dysfunction that impairs autophagic flux [20]. Cardiac endothelial cells likely undergo a similar pathogenic cascade. In this study, CVB3‐induced EndMT was associated with elevated oxidative stress and suppressed autophagy. This paradoxical phenomenon likely reflects a feedback loop between oxidative stress and autophagy. Notably, experimental data indicated a positive correlation between Sirt6 expression and autophagic activity. Sirt6 overexpression partially restored autophagy levels, suggesting its regulatory role in this process. Relevant studies confirm that Sirt6 activation enhances autophagic flux by preventing the accumulation of autophagy‐related factors [46] and ameliorates cardiac hypertrophy via inhibition of the Akt/FoxO3 pathway, which promotes autophagy [47].

In summary, this study demonstrates that Sirt6 inhibits CVB3‐induced EndMT and apoptosis, partially through modulation of oxidative stress and autophagy pathways. Several limitations merit consideration. The conclusions are primarily based on an in vitro model, which may not fully capture the pathophysiology in vivo. Additionally, the precise mechanistic links within the Sirt6‐oxidative stress‐autophagy axis remain to be fully elucidated, and potential functional interactions with other sirtuins have not been investigated. Despite these limitations, our findings provide valuable insights into VMC pathogenesis and identify Sirt6 as a promising therapeutic target for future research.

## Author Contributions

**Zimei Yang:** conceptualization, writing – original draft. **Yimin Wang, Di Zhang**, and **Xinyang Shou:** validation. **Jun Chen** and **Zhidong Zhou:** formal analysis. **Qiang Liu:** writing – review and editing.

## Conflicts of Interest

The authors declare no conflicts of interest.

## Supporting information


**Fig. S1:** Representative immunofluorescence images of MCECs transfected by lentivirus (scale bar: 100μm).

## Data Availability

The authors have nothing to report.

## References

[1] C. Ying , “Viral Myocarditis,” Yale Journal of Biology and Medicine 97, no. 4 (2024): 515–520. 39703606 10.59249/BSHH8575 PMC11650915

[2] Y. Xie , J. Liao , M. Li , et al., “Impaired Cardiac Microvascular Endothelial Cells Function Induced by Coxsackievirus B3 Infection and Its Potential Role in Cardiac Fibrosis,” Virus Research 169, no. 1 (2012): 188–194. 22867880 10.1016/j.virusres.2012.07.027

[3] L. Yang , Q. Liu , Y. Yu , H. Xu , S. Chen , and S. Shi , “Ginsenoside‐Rb3 Inhibits Endothelial–Mesenchymal Transition of Cardiac Microvascular Endothelial Cells,” Herz 44, no. 1 (2019): 60–68. 28983639 10.1007/s00059-017-4628-4

[4] R. S. Machado , F. N. Tavares , I. P. Sousa , and Jr., “Global Landscape of Coxsackieviruses in Human Health,” Virus Research 344 (2024): 199367. 38561065 10.1016/j.virusres.2024.199367 PMC11002681

[5] L. Andréoletti , L. Ventéo , F. Douche‐Aourik , et al., “Active Coxsackieviral B Infection Is Associated With Disruption of Dystrophin in Endomyocardial Tissue of Patients Who Died Suddenly of Acute Myocardial Infarction,” Journal of the American College of Cardiology 50, no. 23 (2007): 2207–2214. 18061067 10.1016/j.jacc.2007.07.080

[6] C. R. Martens and F. Accornero , “Viruses in the Heart: Direct and Indirect Routes to Myocarditis and Heart Failure,” Viruses 13, no. 10 (2021): 1924. 34696354 10.3390/v13101924 PMC8537553

[7] K. S. Kim , G. Hufnagel , N. M. Chapman , and S. Tracy , “The Group B Coxsackieviruses and Myocarditis,” Reviews in Medical Virology 11, no. 6 (2001): 355–368. 11746998 10.1002/rmv.326

[8] H. Jiang , Y. Zhou , W. Zhang , et al., “Molecular Mechanisms of Endothelial–Mesenchymal Transition and Its Pathophysiological Feature in Cerebrovascular Disease,” Cell & Bioscience 15, no. 1 (2025): 49. 40253404 10.1186/s13578-025-01393-y PMC12008988

[9] C. Qian , G. Dong , C. Yang , et al., “Broadening Horizons: Molecular Mechanisms and Disease Implications of Endothelial‐To‐Mesenchymal Transition,” Cell Communication and Signaling 23, no. 1 (2025): 16. 39789529 10.1186/s12964-025-02028-y PMC11720945

[10] Z. Guo , P. Li , J. Ge , and H. Li , “SIRT6 in Aging, Metabolism, Inflammation and Cardiovascular Diseases,” Aging and Disease 13, no. 6 (2022): 1787–1822. 36465178 10.14336/AD.2022.0413 PMC9662279

[11] Y. P. Liu , R. Wen , C. F. Liu , et al., “Cellular and Molecular Biology of Sirtuins in Cardiovascular Disease,” Biomedicine and Pharmacotherapy 164 (2023): 114931. 37263163 10.1016/j.biopha.2023.114931

[12] C. V. Pereira , M. Lebiedzinska , M. R. Wieckowski , and P. J. Oliveira , “Regulation and Protection of Mitochondrial Physiology by Sirtuins,” Mitochondrion 12, no. 1 (2012): 66–76. 21787885 10.1016/j.mito.2011.07.003

[13] N. R. Sundaresan , P. Vasudevan , L. Zhong , et al., “The Sirtuin SIRT6 Blocks IGF‐Akt Signaling and Development of Cardiac Hypertrophy by Targeting C‐Jun,” Nature Medicine 18, no. 11 (2012): 1643–1650. 10.1038/nm.2961 PMC4401084 23086477

[14] K. Wu , Y. Wang , R. Liu , H. Wang , and T. Rui , “The Role of Mammalian Sirtuin 6 in Cardiovascular Diseases and Diabetes Mellitus,” Frontiers in Physiology 14 (2023): 1207133. 37497437 10.3389/fphys.2023.1207133 PMC10366693

[15] S. Xu , M. Yin , M. Koroleva , et al., “SIRT6 Protects Against Endothelial Dysfunction and Atherosclerosis in Mice,” Aging 8, no. 5 (2016): 1064–1082. 27249230 10.18632/aging.100975 PMC4931854

[16] M. Zeng , Z. Chen , Y. Wang , et al., “LncRNA MALAT1 to Enhance Pyroptosis in Viral Myocarditis Through UPF1‐Mediated SIRT6 mRNA Decay and Wnt‐β‐Catenin Signal Pathway,” Cardiovascular Toxicology 24, no. 12 (2024): 1439–1454. 39367210 10.1007/s12012-024-09922-w

[17] Y. Zhang , Y. Dong , Z. Xiong , et al., “Sirt6‐mediated Endothelial‐to‐Mesenchymal Transition Contributes Toward Diabetic Cardiomyopathy via the notch1 Signaling Pathway,” Diabetes, Metabolic Syndrome and Obesity: Targets and Therapy 13 (2020): 4801–4808. 33324079 10.2147/DMSO.S287287 PMC7732976

[18] W. Qin , L. Zhang , Z. Li , et al., “SIRT6‐mediated Transcriptional Suppression of MALAT1 Is a Key Mechanism for Endothelial to Mesenchymal Transition,” International Journal of Cardiology 295 (2019): 7–13. 31399301 10.1016/j.ijcard.2019.07.082

[19] L. Chen , G. Wang , J. He , et al., “Sirt6 Inhibits Endothelial‐to‐Mesenchymal Transition Through Attenuating the Vascular Endothelial Inflammatory Response,” International Immunopharmacology 101, no. Pt B (2021): 108240. 34666304 10.1016/j.intimp.2021.108240

[20] K. Yu , L. Zhou , Y. Wang , et al., “Mechanisms and Therapeutic Strategies of Viral Myocarditis Targeting Autophagy,” Frontiers in Pharmacology 13 (2022): 843103. 35479306 10.3389/fphar.2022.843103 PMC9035591

[21] A. Palabiyik , “The Role of Bcl‑2 in Controlling the Transition Between Autophagy and Apoptosis (Review),” Molecular Medicine Reports 32, no. 1 (2025): 1–9. 10.3892/mmr.2025.13537 PMC12045647 40242969

[22] G. Sahoo , D. Samal , P. Khandayataray , and M. K. Murthy , “A Review on Caspases: Key Regulators of Biological Activities and Apoptosis,” Molecular Neurobiology 60, no. 10 (2023): 5805–5837. 37349620 10.1007/s12035-023-03433-5

[23] T. Fukai and M. Ushio‐Fukai , “Cross‐Talk Between NADPH Oxidase and Mitochondria: Role in ROS Signaling and Angiogenesis,” Cells 9, no. 8 (2020): 1849. 32781794 10.3390/cells9081849 PMC7466096

[24] B. Sun , L. Lin , T. Yao , et al., “Jingfang Granule Mitigates Coxsackievirus b3‐induced Myocardial Damage by Modulating Mucolipin 1 Expression,” Journal of Ethnopharmacology 320 (2024): 117396. 37951374 10.1016/j.jep.2023.117396

[25] J. A. Pan , H. Zhang , H. Lin , et al., “Irisin Ameliorates Doxorubicin‐Induced Cardiac Perivascular Fibrosis Through Inhibiting Endothelial‐to‐Mesenchymal Transition by Regulating ROS Accumulation and Autophagy Disorder in Endothelial Cells,” Redox Biology 46 (2021): 102120. 34479089 10.1016/j.redox.2021.102120 PMC8413906

[26] H. Gong , X. Lyu , Q. Wang , M. Hu , and X. Zhang , “Endothelial to Mesenchymal Transition in the Cardiovascular System,” Life Sciences 184 (2017): 95–102. 28716564 10.1016/j.lfs.2017.07.014

[27] Y. Li , Y. Xiao , Y. Shang , et al., “Exosomes Derived From Adipose Tissue‐Derived Stem Cells Alleviated H2O2‐Induced Oxidative Stress and Endothelial‐to‐Mesenchymal Transition in Human Umbilical Vein Endothelial Cells by Inhibition of the mir‐486‐3p/Sirt6/Smad Signaling Pathway,” Cell Biology and Toxicology 40, no. 1 (2024): 39. 38789630 10.1007/s10565-024-09881-6 PMC11126451

[28] N. N. Tao , J. H. Ren , H. Tang , et al., “Deacetylation of Ku70 by SIRT6 Attenuates Bax‐Mediated Apoptosis in Hepatocellular Carcinoma,” Biochemical and Biophysical Research Communications 485, no. 4 (2017): 713–719. 28238784 10.1016/j.bbrc.2017.02.111

[29] R. Dong , X. Zhang , Y. Liu , et al., “Rutin Alleviates EndMT by Restoring Autophagy Through Inhibiting HDAC1 via PI3K/AKT/mTOR Pathway in Diabetic Kidney Disease,” Phytomedicine 112 (2023): 154700. 36774842 10.1016/j.phymed.2023.154700

[30] L. Zhang , J. He , J. Wang , et al., “Knockout RAGE Alleviates Cardiac Fibrosis Through Repressing Endothelial‐To‐Mesenchymal Transition (EndMT) Mediated by Autophagy,” Cell Death & Disease 12, no. 5 (2021): 470. 33976108 10.1038/s41419-021-03750-4 PMC8113558

[31] L. Zhang , Y. N. Guo , J. Liu , et al., “Plantamajoside Attenuates Cardiac Fibrosis via Inhibiting AGEs Activated‐RAGE/Autophagy/EndMT pathway,” Phytotherapy Research 37, no. 3 (2023): 834–847. 36349468 10.1002/ptr.7663

[32] H. Jie , J. Zhang , S. Wu , et al., “Interplay Between Energy Metabolism and NADPH Oxidase‐Mediated Pathophysiology in Cardiovascular Diseases,” Frontiers in Pharmacology 15 (2024): 1503824. 39867658 10.3389/fphar.2024.1503824 PMC11757639

[33] D. Kračun , L. R. Lopes , E. Cifuentes‐Pagano , and P. J. Pagano , “NADPH Oxidases: Redox Regulation of Cell Homeostasis and Disease,” Physiological Reviews 105, no. 3 (2025): 1291–1428. 39814410 10.1152/physrev.00034.2023 PMC12285607

[34] X. Liu , S. Ren , Z. Li , et al., “Sirt6 Mediates Antioxidative Functions by Increasing nrf2 Abundance,” Experimental Cell Research, 422, no. 1 (2023): 113409. 36356655 10.1016/j.yexcr.2022.113409

[35] Z. Chen , W. Liang , J. Hu , et al., “Sirt6 Deficiency Contributes to Mitochondrial Fission and Oxidative Damage in Podocytes via rock1‐drp1 Signalling Pathway,” Cell Proliferation 55, no. 10 (2022): e13296. 35842903 10.1111/cpr.13296 PMC9528772

[36] S. Gao , Q. Yang , Y. Peng , et al., “SIRT6 Regulates Obesity‐Induced Oxidative Stress via ENDOG/SOD2 Signaling in the Heart,” Cell Biology and Toxicology 39, no. 4 (2023): 1489–1507. 35798905 10.1007/s10565-022-09735-z

[37] G. Chen , F. Zhang , L. Wang , and Z. Feng , “Isoflurane Alleviates Hypoxia/Reoxygenation Induced Myocardial Injury by Reducing miR‐744 Mediated SIRT6,” Toxicology Mechanisms and Methods 32, no. 4 (2022): 235–242. 34663177 10.1080/15376516.2021.1995556

[38] A. Maksin‐Matveev , Y. Kanfi , E. Hochhauser , A. Isak , H. Y. Cohen , and A. Shainberg , “Sirtuin 6 Protects the Heart From Hypoxic Damage,” Experimental Cell Research 330, no. 1 (2015): 81–90. 25066211 10.1016/j.yexcr.2014.07.013

[39] Y. Yang , T. Tian , Y. Wang , et al., “Sirt6 Protects Vascular Endothelial Cells From Angiotensin II‐Induced Apoptosis and Oxidative Stress by Promoting the Activation of nrf2/are Signaling,” European Journal of Pharmacology 859 (2019): 172516. 31265839 10.1016/j.ejphar.2019.172516

[40] A. Kanwal , V. B. Pillai , S. Samant , M. Gupta , and M. P. Gupta , “The Nuclear and Mitochondrial Sirtuins, Sirt6 and Sirt3, Regulate Each Other's Activity and Protect the Heart From Developing Obesity‐Mediated Diabetic Cardiomyopathy,” FASEB Journal 33, no. 10 (2019): 10872–10888. 31318577 10.1096/fj.201900767R PMC6766651

[41] D. Smirnov , E. Eremenko , D. Stein , et al., “SIRT6 Is a Key Regulator of Mitochondrial Function in the Brain,” Cell Death & Disease 14, no. 1 (2023): 35. 36653345 10.1038/s41419-022-05542-w PMC9849342

[42] C. Carresi , R. Mollace , R. Macrì , et al., “Oxidative Stress Triggers Defective Autophagy in Endothelial Cells: Role in Atherothrombosis Development,” Antioxidants (Basel) 10, no. 3 (2021): 387. 33807637 10.3390/antiox10030387 PMC8001288

[43] S. T. Shibutani , T. Saitoh , H. Nowag , C. Münz , and T. Yoshimori , “Autophagy and Autophagy‐Related Proteins in the Immune System,” Nature Immunology 16, no. 10 (2015): 1014–1024. 26382870 10.1038/ni.3273

[44] L. Zhu , Y. Liao , and B. Jiang , “Role of ROS and Autophagy in the Pathological Process of Atherosclerosis,” Journal of Physiology and Biochemistry 80, no. 4 (2024): 743–756. 39110405 10.1007/s13105-024-01039-6

[45] J. Zou , Y. Liu , B. Li , et al., “Autophagy Attenuates Endothelial‐To‐Mesenchymal Transition by Promoting Snail Degradation in Human Cardiac Microvascular Endothelial Cells,” Bioscience Reports 37, no. 5 (2017): BSR20171049. 28811357 10.1042/BSR20171049 PMC5587916

[46] X. Li , L. Liu , W. Jiang , et al., “SIRT6 Protects Against Myocardial Ischemia–Reperfusion Injury by Attenuating Aging‐Related CHMP2B Accumulation,” Journal of Cardiovascular Translational Research 15, no. 4 (2022): 740–753. 35235147 10.1007/s12265-021-10184-y

[47] J. Lu , D. Sun , Z. Liu , et al., “SIRT6 Suppresses Isoproterenol‐Induced Cardiac Hypertrophy Through Activation of Autophagy,” Translational Research 172 (2016): 96–112.e6.e116. 27016702 10.1016/j.trsl.2016.03.002

