# Supplementary material for: Inhibitory Effect of Sirtuin6 on EndMT by Regulating Oxidative Stress and Autophagy in Coxsackievirus B3‐Induced Cardiac Endothelial Cells
Source: Immun Inflamm Dis. 2025 Dec 29;13(12):e70316. doi: 10.1002/iid3.70316 (PMC12748517; doi:10.1002/iid3.70316)
Supplement: Supplementary file 1 — Fig. S1: Representative immunofluorescence images of MCECs transfected by lentivirus (scale bar: 100μm). [file IID3-13-e70316-s001.docx]

**Supplementary material**

**
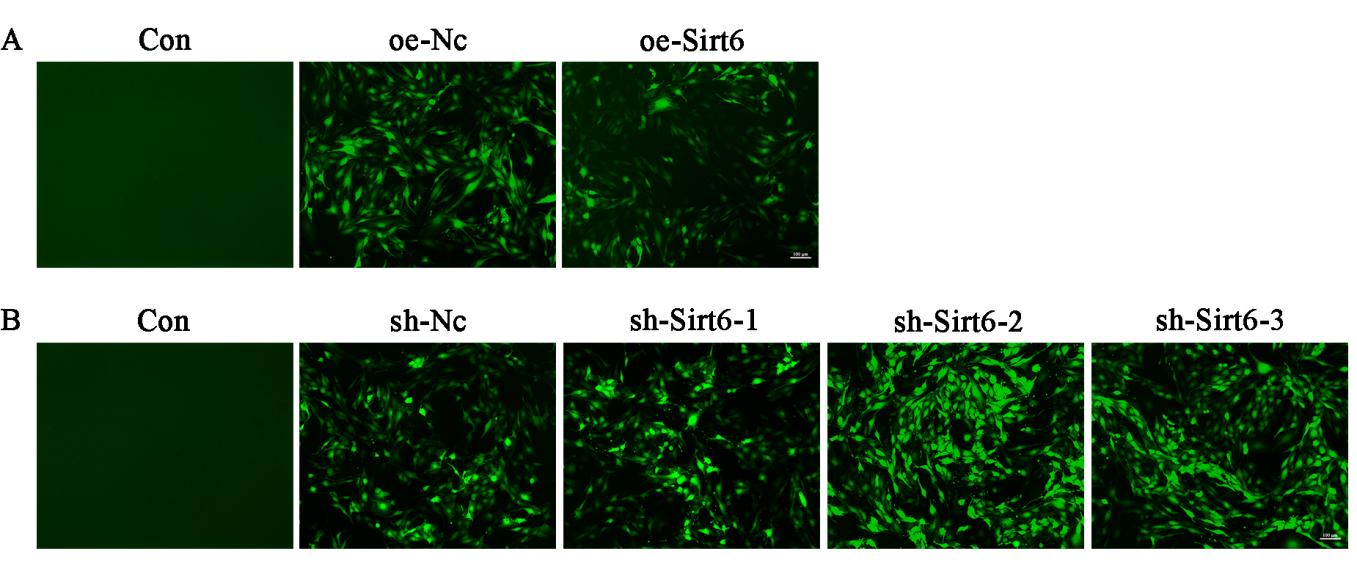
**

Fig. S1 Representative immunofluorescence images of MCECs transfected by lentivirus (scale bar: 100μm).
